# Guiding Young Children’s Digital Media Use: SES-Differences in Mediation Concerns and Competence

**DOI:** 10.1007/s10826-018-1018-3

**Published:** 2018-02-21

**Authors:** Peter Nikken, Suzanna J. Opree

**Affiliations:** 1 Erasmus School of History, Culture and Communication, Erasmus University Rotterdam, P.O. Box 1738, Rotterdam, DR 3000 The Netherlands; 2 Windesheim University of Applied Science, P.O. Box 10090, Zwolle, GB 8000 The Netherlands

**Keywords:** Children, Digital media, Parental mediation, Perceived ease, Perceived competence, Socio-economic status

## Abstract

Previous research about parents’ mediation of their young children’s (digital) media use has predominantly focused on the different types, determinants, and effectiveness of parental mediation strategies. Although research on parents’ perceived mediation concerns and competences is scarce, it is known that, compared to mothers and high-educated parents, fathers and low-educated parents experience greater insecurity (i.e., higher concern and lower competence) when applying media mediation. Based on Bourdieu’s theory of social capital it may be expected that—in addition to educational level—marital status and family income predict parents’ perceived mediation concerns and competences: Family demographics may predict parents’ media proficiency and adoption of new media technologies and these media ecological factors may, in turn, affect perceived concerns and competences. To test this assumption, survey data were collected among 1029 parents of children between the ages of 1 to 9 years. We found that parents’ basic media proficiency was lower in low income, low educated, and single-parent families, whereas parents’ advanced media proficiency was only lower in low educated and single-parent families. As expected, parents’ ease of active co-use was positively associated with parents’ basic proficiency, ease of restrictive mediation by basic and advanced proficiency, and ease of imposing technical restrictions by advanced media proficiency. Parents’ perceived mediation concerns were, however, unrelated to parents’ media proficiency. Also, as expected, low educated parents were less inclined to adopt new media technologies. Adoption of new media was negatively related to perceived mediation concerns, yet did not predict parents’ perceived competence.

## Introduction

Notwithstanding a long tradition in research on parents’ guidance of their children’s media use—focusing on the types, determinants, and effectiveness of parental mediation—we still need more knowledge about parents’ mediation experiences: To what extent do parents have concerns about their mediation practices; how difficult is it for parents to apply media mediation; and finally, which parents experience the greatest concerns and lowest competence and are, hence, in the greatest need of help? Knowledge about parents’ mediation experiences is needed, since guiding children in today’s media environment is an increasingly daunting task for many parents (e.g., Evans et al. 2011; Livingstone et al. 2015). Moreover, following Bourdieu’s (1984) theory of social cultural capital, parents will differ in concerns and mediation practices, since systematic distinctions in social, cultural and economic capital among parents may affect a family’s affinity with media. Therefore, the parents’ and children’s choices for certain types of content, their adoption of media technologies, and their proficiency in using digital devices may vary, along with the parents’ ease of guiding their children in using these media. Children from lower-income, less educated families or with single parents, in particular, may encounter less responsive parental involvement and a less stimulating physical environment (i.e., fewer digital resources at home) than children in more affluent families (Berger et al. 2009; Warren 2005).

Parental mediation consists of all practices by which parents mold and regulate their children’s use of the media, or as Warren (2001) stated ‘any strategy parents use to control, supervise or interpret media content for children’ (p. 212). Parental mediation, thus, entails the mere creation of the child’s media-ecology by acquiring and locating devices at home, as well as applying various practices on their children’s media use. These practices can be divided into restrictive mediation, active mediation, co-use, supervision, monitoring, and the use of technical restrictions (see Nikken and Jansz 2014; Zaman et al. 2016). Research consistently has shown that the frequency by which parents apply mediation strategies and how they create a media environment at home can vary significantly between families. Characteristics of the parents, the children and the family, as well as the parents’ views on the role of media for their child’s development and well-being are important factors for these differences between parents (e.g., Lauricella et al. 2015; Nikken and Schols 2015). Moreover, parental mediation also is transient (Zaman et al. 2016): Parents fluently merge different strategies or hop from one strategy to another depending on the situation at stake. As such, parents’ guidance of their children’s media use is part of the general socialization process in the family, which is defined by overarching parenting norms and styles. An overt communication style (i.e., an open exchange of information among family members), for example, increases a parent’s engagement in media related discussions with their children too (Fujioka and Austin 2002; Warren et al. 2002).

As Livingstone et al. (2015) stated, all parents try to ensure that the use of the media in the home environment matches the parents’ own values and priorities, as well as their children’s needs and media skills. However, at some moment in the process of their parenting chores, parents see themselves confronted with situations that are a concern and may make them feel less competent in their parenting (Rusby et al. 2015). We believe that the recent increase of technologies at home also may pose all kinds of new concerns for parents. A qualitative study by Evans et al. (2011), for example, showed that some parents deliberately avoid restrictions on their child’s television use, because they cannot think of alternative leisure activities or to minimize the risk of quarrels and frustrations in the family. To avoid discussions, some parents also deliberately provide children with media sets in their bedrooms so that the parents can have moments of rest for themselves (Haines et al. 2013; Vaala and Hornik 2014). In similar vein, Livingstone et al. (2015) noted that very apt consumers of modern media technologies experienced problems in restricting their children’s media use, because then they should restrict their own media use too.

In some families, dealing with concerns and doubts about parenting is more difficult than in other families, depending on characteristics of the family, the child, and the parent. For example, less affluent circumstances in the family may affect the quality of parenting, in particular, in terms of sensitivity and responsiveness to the child (McLoyd 1998). Parents may be stressed, depressed, or overworked and therefore less responsive to children’s needs, including less allocation of time and effort to their children’s media use (e.g., Haines et al. 2013; Warren 2005). An explorative study by Nikken and De Haan (2015) showed that parents with children up to 8 years old relatively often experience several problems in guiding their children’s media use. Moreover, 1 in 8 parents reported to be insecure about mediating their children’s media use. Especially fathers encountered somewhat more problems and wanted support more than mothers. Unfortunately, the Nikken and De Haan study only used the parent’s gender and educational level as background characteristics and contained no information about marital status, family income or other family factors. Also, because limited scales were used to measure how parents value daily mediation practices and which concerns they encounter, the authors recommended further research on parental concerns about the mediation of young children’s digital media use.

The extent to which parents have difficulties with mediating children’s media use may vary with situational characteristics of the family. According to Bourdieu’s (1984) theory of social cultural capital, every parent operates within his or her own social field using the resources (i.e., ‘capitals’) available within that field. At least three interrelated and convertible types of capital define how families cope with their daily parenting practices: economic capital, cultural capital, and social capital. Economic capital entails all material properties a person possesses as well as the institutionalized rights and claims one has to such materials, for example, based on the parent’s educational level or income. Cultural capital is formed by the inherited long-lasting dispositions regarding tastes, norms and values, objectified in commodities and consumables, both in a materialistic way (e.g., acquired media technologies at home) and in a symbolic way (e.g., shared understanding and perceived meaning of media content). Cultural capital, as such, also refers to media competencies, skills, and qualifications by which the parent mobilizes his or her cultural authority. Social capital, finally, is defined by all the social obligations, connections, and relationships that a parent has within his or her social domain, for example, other family members, the child’s school or daycare, the local community, the parent’s working environment, or any other specific group based on, for example, race, religion, gender, political view, or a specific interest.

Distinct differences in the social position of families can be linked systematically to variables as education, income, marital status or migrant background, and the prevalence of media devices at home, attitudes about media use, and capacities to make use of the media. Families vary in the ways they can and do make use of support in their social network (i.e., who they turn to, who they believe, and when they ask someone for help). Moreover, families also have inherited specific values from former generations, and these views are compared by parents to current values that are upheld within their social networks: their close friends, next of kin, cultural societies, racial groups, and work environment. Parents thus face both societal and personal pressures, which influence the way they guide young children’s media use (Cingel and Krcmar 2013, p. 380), just like the type of meals they prepare or the type of schools they want their children to attend.

There is ample research which has shown that children have more electronic devices in their bedrooms, are higher screen media consumers, and more infrequent readers when their parents are low in education or socioeconomic status (e.g., Cingel and Krcmar 2013; Gentile and Walsh 2002). Parents low in education or low in income, in particular, also more often prefer to use restrictive mediation as compared to higher educated, wealthier parents (Livingstone et al. 2015; Nikken and Jansz 2006). Higher educated parents, on the other hand, seem more conscious about their mediation. They less often make media a part of baby’s routine (Cingel and Krcmar 2013), more often choose active mediation over restrictive mediation (Livingstone et al. 2015), and confront their young children more with computer technologies, and educational and informative television programming than less wealthy or less educated parents do (Anderson et al. 2001; Calvert et al. 2005). With regard to ethnicity and dominant cultural values, research has also shown that parents differ in how they construct their home environment with media devices, apply different types of parental mediation, and perceive the influence of media on the wellbeing of their children (e.g., Ito et al. 2010; Kirwil 2009). US children from black or Latino/Hispanic minority families, for example, more often are allowed to have a television in their bedroom and can watch more hours per day than Caucasian American children (Gentile and Walsh 2002; Haines et al. 2013). Joint use of digital media, including homework for school with online applications is less normal for Hispanic immigrant parents in the US. This is not so because these families do not own smartphones, tablets or laptops, but because the parents lack basic knowledge to assist their children in using digital media (Katz and Levine 2015). Moreover, children in single-parent homes consume more media, both educational and non-educational, as compared to children in nuclear families (Cingel and Krcmar 2013; Gentile and Walsh 2002).

Distinctions between families in terms of socio-economic or cultural capital may also affect how parents value the role of media for themselves and their children. Lower income families, as compared to higher income families, more often associate media time with family time and, therefore, prefer watching television together over using screens individually (Clark 2012). Other parents, however, may highly value the adoption of the latest high-tech digital media which may induce more feelings of competence in parental mediation. Some parents indeed acquire high-tech devices for their children’s educational benefit or because these devices offer opportunities to engage more deeply in shared play and learning with their children (Chiong and Shuler 2010).

More contemporary screens at home suggests more skills to handle media related problems, because of a longer history of use and more familiarity with the equipment (Huysmans and De Haan 2010) and with that use also more confidence in mediation of children’s media use. According to Austin (1993), the value of parental mediation is indeed predicated on the assumption that adults have mastered the medium to a greater extent than their children have. However, the ongoing adoption and use of technology by children also impacts parents’ responses to their children’s media use. For instance, when young children start to be engaged in social media parents not only increase their active and restrictive mediation (Nikken and Jansz 2014), they also report more problems about their mediation and significantly more often look for professional support (Nikken and De Haan 2015). Also, when children are more skilled in handling devices, parents more often apply all kinds of mediation (Nikken and Schols 2015). On the one hand this implies that parents are more willing to mediate and find it easier to manage children’s media use when they can relate to their child’s capacities in handling digital devices. On the other hand, with more media proficient children, parents may also get more concerned about new risks involved in their children’s media use, and therefore see themselves compelled to adapt their mediation activities.

Given the different capitals that parents can rely on, according to Bourdieu (1984), it is conceivable that parental mediation is not as easy for all parents. With regard to social capital, for example, parenting in families with more children at home and in single-parent families is less easy than in intact families or when there are less children at home. For single parents it is less easy to devote enough time and effort to all children or to give the same attention to firstborns, middle children or the youngest children (Caceres-Delpiano 2006). Single parents, without a sparring partner, may also have more doubt about their parenting practices, find it less easy to maintain rules, and are more in need of parenting support (McLoyd 1998). With more children living at home, parents also may be less restrictive on younger children’s media use (Cingel and Krcmar 2013). The presence of other older children at home is indeed paralleled by more parental concerns about young children’s media use, although more children at home also relates to more competence in mediation and less need for professional support (Nikken and De Haan 2015), perhaps because of earlier experiences with older children’s media use.

With regard to economic capital, it is known that when parents have less possibilities to buy toys, games or high-tech media, the physical environment may offer children less stimulation and fewer resources for healthy development (Berger et al. 2009). Parents may also create more psychological stress in their children as a result of economic insecurity, housing instability or inconsistent employment (Dodge et al. 1994). Less wealthy parents often are less confident in managing online risks too. Lower income parents, in particular those less educated, experience a gap in their digital skills as compared to their children’s, whereas parents in higher-income families are more advanced in adopting and using new technologies and characterized by easily applying a wide range of mediation practices (Livingstone et al. 2015). When there are more screens at home, however, mediation also may become less easy to apply resulting in concerns about the children’s media use. In media saturated homes, parents less often supervise when their children use media devices on their own (Nikken and Schols 2015), and less often exert control and active mediation, or co-use the media with their children (Abelman 2007; Nikken and Jansz 2014).

Concerning a family’s cultural capital, finally, the parent’s education level plays a significant role in how parents manage to apply various mediation practices (e.g., Haines et al. 2013; Nikken and Jansz 2006). Less educated parents often are found to be less consistent in their mediation practices, which may result in more use of media devices by children on their own or the use of inappropriate content at a too young age. Higher-educated parents, on the other hand, usually have less problems in parenting and in managing their children’s media use, although these parents too may encounter media-related concerns (Livingstone et al. 2015).

Using a survey among Dutch parents with children aged 1–9 years, this study expands the recent explorative study by Nikken and De Haan (2015) regarding parents’ concerns about young children’s media activities and parents’ competences in mediation. The main foci of the present study are the extent to which parents experience concerns about their daily mediation (RQ_1_) and the ease/difficulty with which parents apply parental mediation on young children’s media use (RQ_2_). Moreover, we look at how these experiences and competences vary within families – especially in terms of income, parental education and marital status. Based on the discussed theories, we formulated the following seven hypotheses with sub hypotheses, being: There are fewer media devices in the household, but more devices in the child’s bedroom in low income compared to high income families (H_1a_, H_1b_), low educated compared to high educated families (H_1c_, H_1d_), and one-parent versus intact families (H_1e_, H_1f_); Children make more use of entertainment media, but less use of educational media in low income compared to high income families (H_2a_), low educated compared to high educated families (H_2b_), and one-parent versus intact families (H_2c_); Children are less proficient in media use in low income compared to high income families (H_3a_), low educated compared to high educated families (H_3b_), and one-parent versus intact families (H_3c_); Parents are less proficient in media use in low income compared to high income families (H_4a_), low educated compared to high educated families (H_4b_), and one-parent versus intact families (H_4c_); Parents are less inclined to adopt the latest media technologies in low income compared to high income families (H_5a_), low educated compared to high educated families (H_5b_), and one-parent versus intact families (H_5c_); Parental mediation concerns are higher among parents less proficient compared to parents more proficient in media use (H_6a_), and parents less inclined compared to parents more inclined to adopt the latest media technologies (H_6b_); and, finally, perceived ease of mediation is lower among parents less proficient compared to parents more proficient in media use (H_7a_), and parents less inclined compared to parents more inclined to adopt the latest media technologies (H_7b_).

## Method

### Participants

In March 2015 an online questionnaire was presented to more than 5000 parents in the Netherlands who had at least one child between 1 and 9 years at home. The data collection gained IRB-approval from the researchers’ department’s ethical review board. Parents were recruited by a professional market research company. In all, 1029 parents reacted (response rate 20%). After inspection of the data relevant for this study, 248 parents were removed because they did not want to provide their income. The final sample (*N* = 781) contained more mothers (64.8%) than fathers. The marital status of 17.8 percent of the respondents was coded as ‘single-parent’; more often being mothers (21.7%) than fathers (10.5%). Following the Dutch system of educational attainment (OECD, n.d.), more than half of the parents (62.7%) was lower educated (i.e., having a vocational level of education or lower) as compared to higher educated parents (i.e., having a bachelor or master degree or higher). Four in ten parents (42.6%) earned up to 33,000 euro per year, which is somewhat less than the Dutch modal income (34,200 euro; CBS 2015), whereas the other parents earned more than 33,000 euro per year.

### Procedure

In the online questionnaire the parent was asked to answer all questions, keeping a child in mind within the age range of 1 to 9 years who was living at home and who would celebrate his/her birthday first. Completing the questionnaire, on average, took 15 min. The parents reported in almost equal amounts about sons (52.2%) and daughters, and younger and older children: 33.5% had a child between 1 and 3 years in mind; 34.5% a child between 4 and 6 years; and 32% a child between 7 and 9 years. In 4 out of 10 families (39.9%) there were also older children living at home than the child reported on, whereas in a quarter of the families (24.2%) there were also younger children.

### Measures

#### Devices at home

Parents were asked to indicate for eleven media devices how many were present in the home, with the answer options being ‘0’, ‘1’, ‘2’, ‘3’, or ‘4 or more’. Following Nikken and Schols (2015) we created four indexes based on the devices’ handling features. The first index indicates the number of touchscreen operated media (i.e., smartphones, tablets, and children’s tablets; *M = *3.48, *SD* = 1.86); the second index the number of TV media (i.e., televisions, smart-televisions, and Blue ray/DVD-players; *M = *2.80, *SD* = 1.62); the third index the number of mouse/keyboard operated computers (i.e., desktop computers, laptops, and children’s laptops; *M = *2.47, *SD* = 1.47); and the fourth index the number of controller operated consoles (i.e., game consoles and handheld devices; *M = *1.67, *SD* = 1.54). To create these indexes, all answers ‘4 or more’ coded as 4.

#### Devices in the child’s bedroom

If parents indicated that a certain type of device was present in the home and that their child used it regularly, they were asked the follow-up question if the device was present in the child’s bedroom. Here, the answer options were simply (0) no, and (1) yes. For each of the categories presented above we determined whether the child had one or multiple devices in his bedroom, coding this as (0) no, and (1) yes. In total, 10.2% of children had bedroom access to a touchscreen operated medium, 15.0% to a TV medium, 8.6% to a mouse/keyboard operated computer, and 9.4% to a controller operated console.

#### Children’s digital media activities

Parents indicated for 18 different media activities if their child engaged in them and if so, with whom. The answer categories were (1) never, (2) usually alone, (3) usually with the mother, (4) usually with the father, (5) usually with sibling(s), and (6) usually with friends. Examples of activities mentioned in the items are ‘video chatting,’ ‘playing educational games,’ and ‘watching YouTube videos.’ To determine whether the items loaded onto one common factor or multiple factors the answer categories were first recoded into (0) no and (1) yes (i.e., collapsing categories 2–6). Next, we conducted an exploratory factor analysis with oblique rotation (i.e., Quartimin, appropriate for binary variables, see Holmes Finch 2011). The 18 items loaded onto three different factors. The explained variance for the first factor is 34.2%, for the second it is 10.8%, and for the third 7.7%. Only maintaining items with an absolute factor loading of .45 or higher (see Tabachnick and Fidell 2007), the first factor contained five items and captured cognitive and creative use (e.g., ‘playing memory games,’ ‘solving puzzles,’ and ‘drawing’) (loadings ranging from .58 to .80; α = .79; *M = *0.55, *SD* = 0.36); the second factor contained seven items capturing social use (e.g., ‘text messaging’ and ‘using social media’) (loadings ranging from −0.86 to −0.48; α = 0.83; *M = *0.28, *SD* = 0.31), and the third factor contained five items capturing entertainment use (e.g. ‘playing adventure games’ and ‘playing shooting games’—including educational games as well) (loadings ranging from 0.47 to 0.79; α = 0.73; *M = *0.60, *SD* = 0.32).

#### Children’s media proficiency

Children’s media proficiency was measured as parents’ agreement with 25 items about their children’s media skills. Each item contained a statement like ‘my child can use the mouse or keyboard appropriately, for example when playing a game,’ ‘my child can find his/her favorite websites by itself,’ or ‘my child understands that video clips on TV or the internet may be unreal.’ Parents were asked to indicate whether or not the statement applied to their child, and could specify their answer on a 5-point Likert scale. The possible answers were (1) fully inaccurate, (2) inaccurate, (3) not inaccurate nor accurate, (4) accurate, and (5) fully accurate. An exploratory factor analysis with oblique rotation (i.e., direct Oblimin) was conducted to determine whether the 25 items shared one common factor or loaded on multiple factors. The 25 items loaded onto two different factors, the explained variance of the first factor (65.7%) greatly exceeding that of the second (5.8%). Only maintaining items with an absolute factor loading of .45 or higher and removing two items with double loadings, the first factor contained 19 items and captured children’s basic and advanced technical skills as well as understanding media (loadings ranging from .45 to .96); the second factor contained three items that seemingly did not share a common theme and is omitted from this study. We created a scale score for children’s media proficiency by averaging scores on the items of the first factor (α = 0.97; *M = *3.33, *SD* = 1.09).

#### Parent’s media proficiency

Parents’ media proficiency was measured as parents’ rating of their own media skills, using 16 items based on Correa et al. (2015). Each item contained a skill like ‘uploading a music- or video-file,’ ‘installing an anti-virus program on the computer,’ or ‘changing a ringtone on a smartphone.’ Parents rated their proficiency on a 5-point Likert scale, with the answer options being ‘I find this…’ (1) very difficult, (2) difficult, (3) not difficult nor easy, (4) easy, and (5) very easy. Parents could also select a sixth option, being ‘I have never done this’. If parents chose this option, the behavior cannot be rated as difficult nor easy and, for this reason, these responses were recoded as 3’s. An exploratory factor analysis with direct Oblimin rotation was conducted to determine whether the 16 items shared one common factor or loaded on multiple factors. The 16 items loaded onto two different factors, the explained variance of the first factor (62.9%) greatly exceeding that of the second (7.2%). The first factor contained nine items that captured basic technical skills (e.g., searching for an image online or creating an e-mail receiver list) (loadings ranging from 0.57 to 1.00; α = .95; *M = *4.24, *SD* = 0.76); the second factor contained seven items that captured advanced technical skills (e.g., updating the operating system or setting parental controls) (loadings ranging from 0.48 to 0.98; α = .91; *M = *3.89, *SD* = 0.79). We created subscale scores by averaging parents’ score on each factor’s designated items.

#### Parent’s adoption of ICT

To measure parent’s adoption of ICT, they indicated to what extent they agreed with seven statements about acquiring or using the latest high-tech media, for example, ‘I like to be aware of the newest technological inventions like *Google glass* or the *Oculus rift*,’ ‘I think it’s important to be the first to use media-platforms like *Netflix*,’ and ‘when there is a new version of the smartphone, I want to have it.’ The answering options varied on a 5-point Likert scale being (1) fully disagree, (2) disagree, (3) do not disagree nor agree, (4) agree, and (5) fully agree. An exploratory factor analysis with direct Oblimin rotation was conducted to determine whether the seven items shared one common factor – which they in fact did. The explained variance for this factor is 60.3%, and loadings ranged from .55 to 90. We created a scale score for parent’s adoption of ICT by averaging parents’ scores on all seven items (α = 0.90; *M = *2.52, *SD* = 0.84).

#### Parental mediation concerns

Parental concerns about mediation were measured with a set of 16 media-related pedagogical concerns, for example, ‘How can I determine whether a website, app, or game is appropriate for my child?’ or ‘How old should my child be before he/she can have a social media account?’ To measure the prevalence of these concerns in the family parents indicated on a 5-point Likert scale to what extent they worried about a concern. The answer options were (1) not at all, (2) not really, (3) sometimes, (4) regularly, and (5) often. Of the 16 concerns, eleven were taken from Nikken and De Haan (2015) and five originated from the website *mediaopvoeding.nl* where parents can consult experts if they have questions about the use of media by their children. An exploratory factor analysis with direct Oblimin rotation showed that the 16 items loaded on one factor, explaining 62.9% of the variance (loadings ranging from .73 to .84). The 16 items were, therefore, averaged into one scale (Cronbach’s α = 0.96; *M = *2.34, *SD* = 0.80).

#### Ease of parental mediation

Ease of parental mediation was measured with 29 items about parental mediation. For each mediation activity, parents indicated the level of difficulty in applying it on their child’s media use. The activities related to restrictive and active mediation, supervision, co-use, and monitoring (Livingstone and Helsper 2008; Nikken and Schols 2015), for example, deciding whether a child is allowed to have his/her own television set or tablet, monitoring children’s media use, and talking about online risks. Again, a 5-point Likert scale was used, anchored by (1) very difficult and (5) very easy. Parents could also select a sixth option, ‘Not applicable,’ in case a certain activity is irrelevant to them. These responses were recoded as 3’s, since irrelevant makes the application of that mediation not difficult nor easy. An exploratory factor analysis with direct Oblimin rotation was conducted to determine whether the 29 items shared one common factor or loaded on multiple factors. The 29 items loaded onto three different factors, and the explained variance of the first factor (49.2%) greatly exceeds that of the second (4.3%) and third (4.2%). Only maintaining items with an absolute factor loading of .45 or higher, the first factor contained thirteen items and captured both active mediation and co-use activities, thus representing active co-use (cf. Livingstone and Helsper 2008; loadings ranging from .51 to .83; α = .94; *M = *3.68, *SD* = 0.60); the second factor contained five items that captured restrictive mediation (loadings ranging from .51 to .77; α = .83; *M = *3.69, *SD* = 0.68); and the third factor contained three items that captured technical restrictions (loadings ranging from 0.67 to 0.79; α = 0.81; *M = *3.49, *SD* = 0.74). We created subscale scores by averaging parents’ score on each factor’s designated items.

### Data Analyses

To examine differences between low income versus high income, low educated versus high educated, and one-parent versus intact families, independent-samples t-test were conducted for the number of media devices in the household (H_1a_, H_1c_, H_1e_), children’s use of entertainment media (H_2_), and children’s media proficiency (H_3_). The results of these tests are presented in Table 1. This table also contains the results of chi-square tests of independence looking into the difference between low income versus high income, low educated versus high educated, and one-parent versus intact families in the percentage of children that have touchscreens, TV sets, computers, and game consoles in their bedroom (H_1b_, H_1d_, H_1f_). Finally, Table 1 also contains the results of the independent-sample t-tests for parents’ media proficiency (H_4_) and parents’ adoption of ICT (H_5_).

**Table 1:** Media Device Ownership, Children’s Media Use, Children’s and Parents’ Media Proficiency and Parental Attitudes Toward ICT’s by the Parents’ Income, Education and Marital Status

	Income		Educational level		Marital status		Total	
	Low	High	Low	High	Single	Intact	Mean	SD
# Devices at home								
Touchscreens	3.02	3.82***	3.31	3.76***	2.90	3.60***	3.48	1.86
TV sets	2.65	2.92*	2.77	2.86	2.81	2.80	2.80	1.62
Computers	2.38	2.54	2.33	2.71***	2.28	2.51	2.47	1.47
Game consoles	1.59	1.73	1.66	1.68	1.62	1.68	1.67	1.54
% Households with in child’s room…								
Touchscreens	11.7	9.2	10.2	10.3	17.3	8.7***	10.2	3.0
TV sets	17.4	13.2	15.9	13.4	24.5	12.9***	15.0	3.6
Computers	10.2	7.4	7.6	10.3	12.2	7.8^†^	8.6	2.8
Game consoles	8.7	9.8	8.8	10.3	10.1	9.2	9.4	2.9
Children’s media use								
Cognitive/creative use	0.56	0.55	0.55	0.57	0.61	0.54^†^	0.55	0.36
Social use	0.31	0.26†	0.26	0.32*	0.39	0.26***	0.28	0.31
Entertainment use	0.61	0.59	0.60	0.61	0.67	0.58**	0.60	0.32
Children’s media proficiency	3.29	3.35	3.37	3.25	3.60	3.27***	3.33	1.09
Parents’ media proficiency								
Basic media skills	4.15	4.30**	4.20	4.31^†^	4.12	4.26†	4.24	0.76
Advanced media skills	3.85	3.93	3.84	4.00**	3.78	3.92†	3.89	0.79
Parents’ adoption of ICT	2.52	2.51	2.45	2.63**	2.53	2.51	2.52	0.84

^†^*p* < .100; *p < .050; **p < 0.01; ***p < .001

The regression analyses that were used to test whether parental media concerns are associated with parents’ media proficiency (H_6a_) and their inclination to adopt the latest media technologies (H_6b_), as well as whether perceived ease of mediation depends on parents’ media proficiency (H_7a_) and their inclination to adopt the latest media technologies (H_7b_), are presented in Table 2. In order to control for confounding relationships between the characteristics of the parents, the family and the children, hierarchical OLS regression analysis was used, enabling us to determine the relative impact of the parents’ media proficiency and inclination to adopt the latest media technology. A 3-step model was used with income, education, marital status and the parent’s gender as predictors in the first step. In the second step, domestic factors were entered, including the presence of siblings and of media devices at home. In the third step the child’s and parent’s proficiency in media use and the parent’s adoption of new media were entered together with the child’s engagement with media content and gender. Also, the parent’s concerns about their children’s media use was used as a predictor of the ease of parental mediation. Since a test for collinearity showed that the age of the child and the proficiency to use media were highly interrelated (cf. Nikken and Schols 2015), the child’s age was dropped from the list of predictors in all analyses.

**Table 2:** Hierarchical Regression Predicting of Mediation Concerns and Ease of Parental Mediation by Parent, Family, and Child Characteristics and Parental Attitudes (Standardized Coefficients)

	Parental mediation concerns			Ease of active mediation and co-use			Ease of restrictive mediation			Ease of imposing technical restrictions		
Income	−0.05	−0.05	−.03	.07^†^	0.05	−0.00	0.14***	0.11**	0.06^†^	0.10*	0.07^†^	0.06
Education level (0 = low; 1 = high)	0.01	−0.00	−0.01	−0.05	−0.06	−0.05	−0.03	−0.04	−0.03	−0.05	−0.07^†^	−0.08*
Marital status (0 = single, 1 = intact)	−0.18***	−0.17***	−0.13***	−0.01	−0.03	0.00	−0.04	−0.06	−0.03	−0.03	−0.05	−0.03
Gender (0 = father; 1 = mother)	−0.20***	−0.15***	−0.08*	0.12***	0.12***	0.08*	0.12***	0.14***	0.09**	−0.01	0.01	0.03
Other younger children at home^1^		0.03	0.03		−0.01	−0.06^†^		0.01	−0.03		−0.03	−0.06*
Other older children at home^1^		−0.01	0.01		−0.03	−0.03		0.01	0.01		−0.03	−0.02
# Touchscreens at home		0.06	0.03		0.14**	0.04		0.13**	0.06		0.20***	0.11**
# TV sets at home		−0.03	−0.04		−0.03	0.04		−0.00	0.06		−0.10*	−0.04
# Computers at home		0.02	−0.01		0.03	−0.02		0.06	0.03		0.09^†^	0.02
# Consoles at home		0.03	0.02		0.04	0.04		0.02	0.01		0.05	0.05
Touchscreens in bedroom^1^		0.07†	0.03		−0.08*	−0.05		−0.06	−0.02		−0.07^†^	−0.04
TV sets in bedroom^1^		0.08†	0.08*		0.02	−0.00		0.05	0.03		0.05	0.02
Computers in bedroom^1^		0.09*	0.04		−0.05	0.00		−0.08†	−0.03		−0.04	0.01
Consoles in bedroom^1^		0.06	0.03		0.02	−0.00		0.04	0.02		.04	0.02
Gender (0 = boy; 1 = girl)			−0.02			0.03			−0.02			0.01
Cognitive / creative media use			0.08†			0.13***			0.03			0.04
Social media use			0.02			−0.02			0.03			−0.02
Entertainment media use			0.00			−0.08			−0.06			−0.04
Children’s media proficiency			0.08†			0.32***			0.30***			0.21***
Parents’ basic media skills			−0.10			0.35***			0.22***			0.05
Parents’ advanced media skills			−0.07			0.08			0.14*			0.41***
Adoption of ICT			0.29***			−0.03			−0.09*			0.01
Parental mediation concerns						−0.05			−0.08*			0.01
Adjusted *R*^*2*^	0.06	0.10	0.20	0.02	0.03	0.34	0.02	0.05	0.26	0.00	0.05	0.31
F	14.21***	7.24***	9.57***	3.96**	2.72***	18.32***	5.76***	3.75***	12.98***	1.74	3.72***	15.88***

^ †^*p* < 0.100; *p < 0.050;**p < 0.010; ***p < 0.001. ^1^ 0 = no; 1 = yes

## Results

### Differences in Parenting and Children’s Access and Experience with Media

H_1a_ proposed that there would be fewer media devices in low income compared to high income families. This expectation was confirmed for touchscreens (*M = *3.02 vs. *M = *3.82; *p = *0.000) and TV sets (*M = *2.65 vs. *M = *2.92; *p = *0.028), but not for computers and game consoles (i.e., *p = *0.142 and *p = *.212). Similarly, H_1c_ proposed that there would be fewer media devices in low educated compared to high educated families. This expectation was confirmed for touchscreens (*M = *3.31 vs. *M = *3.76; *p = *.001) and computers (*M = *2.33 vs. *M = *2.71, *p = *0.001), but not TV sets and game consoles (*p = *0.438 and *p = *0.904). Finally, H_1e_ proposed that there would be fewer media devices in single-parent compared to intact families. This expectation was only confirmed for touchscreens (*M = *2.90 vs. *M = *3.60, *p = *.001), and not for TV sets, computers, and game consoles (*p = *.952, *p = *0.105, and *p = *0.675). In sum, low income, low educated, and/or single-parent families own fewer touchscreens than high income, high education, and/or intact families. However, just as in high income, high education, and/or intact families, the low income, low educated, and/or single-parent families own more touchscreen devices than any other type of media device.

H_1b_, H_1d_, and H_1f_ proposed that there would be fewer media devices in the bedrooms of children from low income compared to high income families, low educated versus high educated families, and single-parent versus intact families. H_1b_ and H_1d_, pertaining to income and educational level, were rejected for all media devices, but the chi-square tests of independence reveal that children in single families are twice as likely to have a touch screen or TV set in their bedroom (17.3% versus 8.7%, and 24.5% versus 12.9%), and about 1.5 times as likely to have a computer in their bedroom (12.2% versus 7.8%), confirming H_1f_.

H_2_ stipulated that children’s use of entertainment media would be higher in low income than in high income families (H_2a_), in low educated than in high educated families (H_2b_), and in single-parent than in intact families (H_2c_). As can be derived from Table 1, H_2a_ and H_2b_ have to be rejected because no significant effects were found (*p* = 0.603 and *p = *0.629). H_2c_ on marital status, however, was confirmed (*M = *0.67 vs. *M = *0.58, *p = *0.002). Looking at the other types of media use, it is striking that children in single-parent families were also more likely to use media for cognitive and creative purposes (*M = *0.61 vs. 0.54; *p = *0.054), and for social purposes (*M = *0.39 vs. *M = *0.26; *p = *0.000). Since creative and cognitive media use, social media use, and entertainment use in children from single-parent families exceeds that of children from intact families, it may be derived that children from single-parent families use more media in general.

H_3_ suggested that children’s media proficiency would be lower in low income than in high income families (H_3a_), in low educated than in high education families (H_3b_), and in single-parent than in intact families (H_3c_). Again, no differences were found between children from low income versus high income families (*p = *0.423) and between children from low educated versus high educated families (*p = *0.149), resulting in the rejection of H_3a_ and H_3b_. Differences were found between children from single-parent versus intact families, but in the opposite of the hypothesized direction: Children from single-parent families were actually more media proficient than children from intact families (*M = *3.60 vs. *M = *3.27; *p = *0.001).

### Differences in Attitudes toward Media and Parents’ Media Use and Adoption

H_4_ and H_5_ related to differences in, respectively, parents’ media proficiency and parents’ inclination to adopt the latest media technologies. Differences between low and high income (H_4a_, H_5a_), low and high educated (H_4b_, H_5b_), and single-parent and intact families (H_4c_, H_5c_) were examined. H_4_ was tested for parents’ basic and parents’ advanced media skills separately. H_4a_, H_4b_, and H_4c_ were all confirmed for the basic media skills: Generally, compared to high income, high educated, and/or intact families, parents’ basic media skills were lower in the low income (*M = *4.15 vs. 4.30; *p = *0.007), the low educated (*M = *4.20 vs. *M = *4.31; *p = *0.055), and the single-parent families (*M = *4.12 vs. *M = *4.26; *p = *0.085). For parents’ advanced media skills H4_a_ had to be rejected, because no difference in skills between parents from low income and parents from high income families was found (*p = *.154). Still, differences were found in parents’ advanced media skills between low educated and high educated families (*M = *3.84 vs. *M = *4.00; *p = *0.009) and between single-parent and intact families (*M = *3.78 vs. 3.92; *p = *0.097), confirming H_4b_ and H_4c_. Regardless of income, educational level, and marital status, parents’ basic media skills generally – and understandably – exceeded their advanced media skills (*M = *4.24 vs. *M = *3.89). Notably, in all cases, parents’ media proficiency exceeded that of their children.

With regard to parents’ inclination to adopt the latest media technologies, no differences were found between low income and high income families (*p = *0.872) and between single-parent and intact families (*p = *.853), rejecting H_5a_ and H_5c_. However, as expected, parents from low educated families were less inclined to adopt new media technologies than parents from high educated families (*M = *2.45 vs. *M = *2.63; *p = *0.002).

### Perceived Difficulties in Parental Mediation

To answer our research questions (i.e., RQ_1_: To what extent do parents have concerns about their children’s daily media use, and RQ_2_: How easy or difficult is it for parents to apply mediation to their children?), we will first describe parents’ initial reactions towards the questions about parental mediation concerns and ease of mediation. Next, we present the results of a series of regression analyses, performed to determine the relative relationship between income, education, and marital status and the level of parental mediation concerns (H_6_) and the perceived ease of parental mediation (H_7_).

**Fig. 1:**
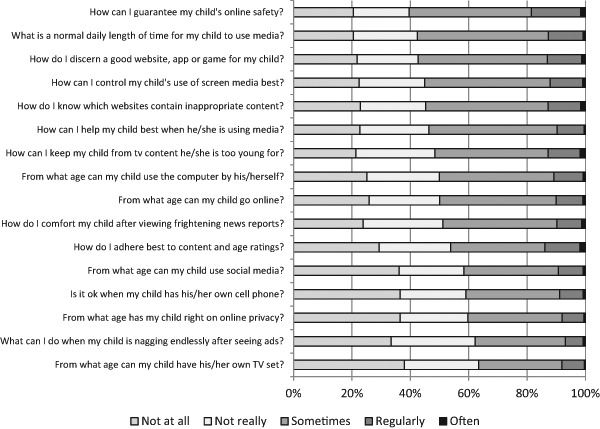
Extent to which potentially media-related concerns are at stake in the parent’s mediation practices

The extent to which parents encounter troubling situations in the daily upbringing of their children differs greatly between parents (*M = *2.34, *SD = *0.80). Like its original items, the scale for perceived difficulty in parental mediation ranged from (1) not at all to (5) very much. Of all parents, 36.1% had a score between 1 and 2 and 49% had a score between 2 and 3. Though only 14.9% had a score above 3, indicating concerns about their child and media use, it is wrong to conclude that parents hardly perceive any difficulties in parental mediation. Figure 1 depicts parents’ scores on each of the 16 individual items. For each troubling situation, the number of parents indicating that it is at least “somewhat” at stake ranges between 37.0 and 60.4%. Concerns that occupy parents the most are guaranteeing the child’s online safety (60.4%), determining the appropriate daily amount of media time (57.6%), discerning good websites, apps or games for children (57.4%), controlling a child’s use of screen media (55.2%), knowing how which websites contain inappropriate content (54.8%), and being able to help the child when it is using media (53.8%). As many as nine out of the sixteen situations troubled at least 50% of all parents.

**Fig. 2:**
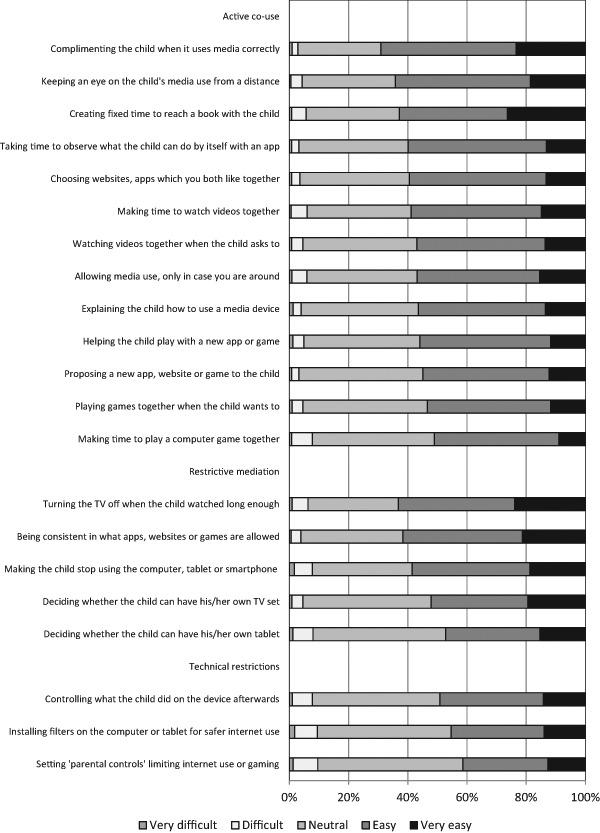
Extent to which parents rate three types of parental mediation activities as ‘difficult’ or ‘easy’ to apply on their child’s media use

Like the perceived concerns, the perceived ease of mediation differed greatly among parents too. We used three scales to measure perceived ease of mediation (i.e., active co-use, restrictive mediation, and technical restrictions), each consisting of multiple questions about specific parental mediation practices for which the parents rated their perceived ease on a scale ranging from (1) very difficult to (5) very easy. Looking at active co-use (*M = *3.68, *SD = *0.60), 0.8% of parents had a score between 1 and 2 and 19.7% had a score between 2 and 3. In other words, 1 in 5 parents struggled with active co-use. With restrictive mediation (*M = *3.69, *SD = *0.68), 0.9% of parents had a score between 1 and 2 and 24.6% had a score between 2 and 3. Hence, 1 in 4 parents struggled with restrictive mediation. Finally, with technical restrictions (*M = *3.45, *SD = *0.74), 3.2% of parents had a score between 1 and 2 and 40.1% had a score between 2 and 3. Thus, more than 4 in 10 parents struggled with imposing technical restrictions. Still, it is important to note that parents’ struggles on all three dimensions seem to be the result of accumulated smaller issues. As Fig. 2 depicts, each separate activity is listed as ‘very difficult’ or ‘difficult’ by no >9.5%.

As Table 2 shows, income, education and marital status are of little importance for the parental concerns and the ease of mediation; in the first models only 0–6% of the variance is explained. The domestic predictors in the second models also have little influence on the additional amount of variance explained (1–5%). The child’s media use and the parent’s views on media in the third models, however, made an important contribution to the total amount of the variance explained (10% increase for parental concerns and 21–31% extra variance for the parent’s ease of mediation). Still, looking at the results from Table 2, it becomes apparent that H_6a_ has to be rejected: Both parents’ basic and advanced media skills did not predict parental mediation concerns (*p = *0.115 and *p = *0.270). H_6b_, in contrast, was confirmed: The more inclined parents are to adopt the latest media technologies, the less parental mediation concerns they experience; meaning that, reversely, the less inclined parents are to adopt the latest media technologies, the more mediation concerns they experienced (*p = *0.000).

H_7a_ (i.e., the less media proficient parents are, the lower their perceived ease of mediation or, reversely, the more media proficient parents are, the higher their perceived ease of mediation) yielded mixed results between the types of mediation and the types of skills. Ease of active co-use was predicted in the proposed direction by parents’ basic skills (*p = *0.000), but not their advanced skills (*p = *0.164); ease of restrictive mediation by both parents’ basic (*p = *0.000) and advanced skills (*p = *0.025); and, finally, ease of imposing technical restrictions was not predicted by parents’ basic skills (*p = *0.347), but was predicted by their advanced skills (*p = *0.000).

H_7b_ (i.e., the less inclined parents are to adopt the latest media technologies, the lower their perceived ease of mediation or, reversely, the more inclined parents are to adopt the latest media technologies, the higher their perceived ease of mediation) was rejected for all forms of mediation. For the ease of active co-use and for the ease of imposing technical restrictions no significant effects were found (*p = *0.435 and *p = *0.835), and for ease of restrictive mediation the effect was in the opposite direction (*p = *0.026). In other words, the more inclined parents were to adopt the latest media technologies, the lower their perceived ease of restrictive mediation.

## Discussion

Expanding a recent study by Nikken and De Haan (2015), the present study aimed to establish the extent to which parents with children aged 1 to 9 years have concerns about their children’s media use (RQ_1_) and how they feel about their competence in mediation practices (RQ_2_). In addition, we were interested whether systematic differences exist between high and low income families, high and low educated families, and single-parent versus intact families regarding the presence and use of media at home, technology adoption and proficiency in media use, and the parent’s mediation competences and concerns in these homes (H_1_–H_5_). Also, we tested relationships between the parent’s proficiency in using these media and adoption of technology with their competences and concerns (H_6_–H_7_). Research on parental guidance of young children’s use of technologies is important, because there is still little systematic information on how parents value the mediation of their children’s media use. Moreover, children’s media use routines, cultural tastes and preferences are shaped already at a young age and knowledge about which families are most in need may lead to better targeted programs or interventions that can assist parents in their mediation activities.

From our findings we may, first, conclude that the majority of parents on average are not very bothered by concerns about their young child and the media at home (RQ_1_). Also, most parents on average are positive about their co-active and restrictive mediation activities, and their application of technical restrictive measures (RQ_2_). However, up to half of the parents do report that one or more individual concerns about mediation prevail rather often in their daily childrearing (cf., Nikken and De Haan 2015). Also, around 10 percent of the parents report that applying mediation is difficult or very difficult, whereas another substantial portion of the parents is ambiguous about the difficulty or easiness of mediation. In other words, among Dutch parents, guiding young children’s use of electronic screens apparently is not an easy task for every parent.

Parent’s media proficiency was not related to the prevalence of concerns about children and media (H_6a_). Given the fact that lower income parents, lower educated parents and single parents all reported lower basic or advanced media proficiency, specifically in these families parental mediation may be more difficult when these parents have less skills in handling the media. Moreover, in an absolute sense, marital status showed to be one of the most consistent background factors for how children engage with media at home, as compared to income and educational level. Although single-parent families have less touchscreens at home, young children in these families still have significantly more electronic devices in their room, make more use of most types of media content, and are reported to be more proficient in handling these devices as compared to children in intact families, whereas their parents are somewhat less proficient. Thus, in single-parent families the gap in media proficiency between parents and their young children seems to be the greatest, which may explain that concerns about children and media are more often recognized by single parents as compared to parents from intact families.

Single parents are known to experience more troubles in parenting in general (Caceres-Delpiano 2006; McLoyd 1998), which makes it understandable that they also have less opportunities to guide their children’s media consumption and give their children more freedom to use media devices on their own. Perhaps single parents have more concerns, but do not report more difficulties in mediation, just because when children use media on their own this provides a moment of rest for their single parents (cf. Evans et al. 2011; Haines et al. 2013). Yet, as the results have shown, young children in single-parent homes are more interested in social media and entertainment media, increasing the risk of inappropriate content and contacts. With Warren (2005) who noticed a decade ago already for television mediation, we conclude that single parents may have a higher need for parenting support in order to cope with media. Parenting support could focus at how parents can mediate different media content types for young children, and/or teach them how to use new media technologies themselves. As we surmised, along with the child’s proficiency in media use, the parent’s own proficiency strongly paralleled their ease of applying mediation (H_7a_). In families where both parents and children have more confidence in handling the media, mediation poses less of a burden on the parent.

We presumed that the parents’ media adoption of new media technologies would affect their mediation competences and concerns (H_6b_, H_7b_). In families less inclined to adopt new media technologies parents indeed have more concerns about children and media and also more easily apply restrictions on their children’s media use. This may indicate that parents who are reluctant on using the latest media technologies, are less skilled themselves and perhaps more afraid about media, and therefore just simply ban media for their young children. Avid technology parents, on the other hand, like Livingstone et al. (2015) noted, worry more about how to restrict media use for their young children, while maintaining their own media habits. Perhaps a higher understanding of media among these parents induces specific thoughts about the risks and benefits of new technologies for their children, which may make the mediation for them extra hard. Our study cannot explain why these parents are fond of acquiring new technologies, but advertising by the media industry or social pressure from friends might play a role. Future studies could thus focus on how parents deal with external pressures in relationship to their parenting chores and parental mediation activities.

Our research questions and assumptions were guided by Bourdieu’s (1984) theory of social cultural capital, that states differences in the quality of parenting are determined by the resources a parent has in his/her social field, in particular social, economic and cultural resources. We expected that family characteristics like education level, income, and marital status, affect the prevalence of media devices at home and the actual media use and media skills of family members (H_1_-H_5_). As discussed above, marital status indeed determines how media are used at home by the children, and whether concerns about children and media prevail among these parents. In same vein, a lower family income and, to a lesser extent, a lower educational level also are both associated with less confidence in applying restrictive measures among parents on their young children’s media use. However, these relationships become irrelevant when the parent’s attitude about new technologies and the parent’s and child’s proficiency in media use are taken into account. In other words, a family’s socio-economic resources may indeed determine how parents guide the child’s media use at home, but the way family members approach media proficiently, as a cultural capital, is much more important for how parental mediation takes place.

The fact the parent’s proficiency in media use parallels more competence in mediation, may indicate that media literate parents probably are more aware of the beneficial or benign outcomes of certain types of media content. Therefore, they know better how to guide their children in using specific devices, like touchscreens, as compared to less literate parents. Moreover, the ease by which parents apply parental mediation on their child’s media use also is strongly tied to the child’s media skills (cf. Nikken and Schols 2015), and to some extent to the types of media content that the child uses. Probably, with media skilled children parents are better able to communicate about the media and to share experiences based on their own media literacy, than with children who do not yet have the capacities to use the media in a self-reliant manner. Future studies could thus expand on our results and take the family members’ proficiency to handle the media more often into account when measuring parental mediation.
